# Histamine: A key compound in red light‐enhanced *Fusarium verticillioides* resistance in maize

**DOI:** 10.1002/imt2.70020

**Published:** 2025-04-07

**Authors:** Xuanjun Feng, Dan Zheng, Weixiao Zhang, Huihui Xiao, Huarui Guan, Hao Xiong, Li Jia, Xuemei Zhang, Wenming Wang, Haiyang Wang, Yanli Lu

**Affiliations:** ^1^ State Key Laboratory of Crop Gene Exploration and Utilization in Southwest China Sichuan Agricultural University Wenjiang China; ^2^ Maize Research Institute Sichuan Agricultural University Wenjiang China; ^3^ State Key Laboratory for Conservation and Utilization of Subtropical Agro‐Bioresources South China Agricultural University Guangzhou China

## Abstract

In this study, we demonstrate that red light is the most critical light component for promoting healthy maize growth during *Fusarium verticillioides* infection. Red light receptors *PHYTOCHROME B* (*PHYB*) and *C* (*PHYC*) play essential roles in maize defense against this pathogen. Overexpression of *PHYC* in maize enhances resistance to *F. verticillioides*. Additionally, we identified two defense‐related gene networks and some metabolites that reliant on *PHYC*s, involving key contributors such as *WRKY* transcription factors and metabolites like histamine and thiamine. Notably, the application of 50 μM histamine significantly boosts resistance, particularly under high‐density conditions, marking the first report of the role of histamine in disease resistance in plants.

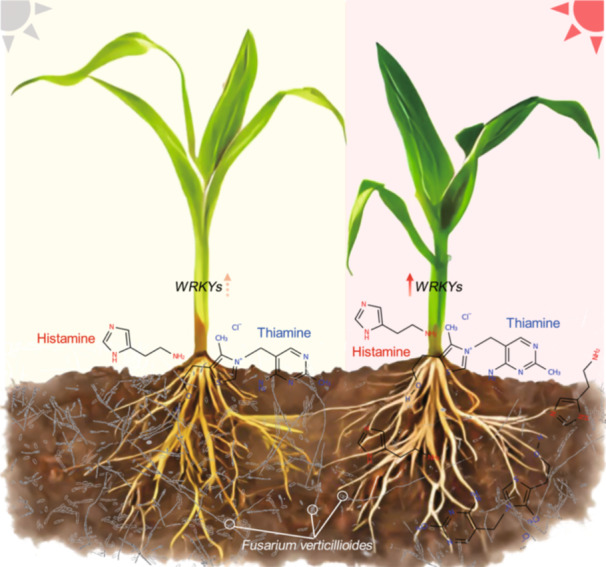

To the Editor,

Light signal is an important factor for plant growth and adaptation to environmental stress [1, 2, 3]. Red, blue, and ultraviolet light have been mostly reported to boost plant defenses [2, 3, 4, 5]. Conversely, far‐red light usually reduces the plant's defenses [2, 3, 6, 7]. The mutual shading of the leaves significantly reduces the ratios of red to far‐red (R:FR), blue to far‐red (B:FR), and ultraviolet to far‐red (UV:FR) light, as well as the overall light intensity under the canopy, thereby facilitating the onset of diseases (Figure S1A‒F). Dense planting is an effective agronomic practice for increasing yield, particularly for maize [8]. However, maize stalk rot caused by *Fusarium verticillioides* was dramatically more severe in the high‐density condition than in the normal‐density condition (Figure S1A,D,E).

We found that red light, rather than far‐red, blue, or UV light, significantly enhanced maize resistance to *F. verticillioides* (Figure S1). Furthermore, supplementation of red light under the light intensity similar to that under the in‐field canopy can effectively improve the resistance of maize to this pathogen (Figure S2). Please read the supplementary results for more details about this part. Therefore, we hope to explore the following questions: (1) whether the resistance of maize to *F. verticillioides* can be improved by manipulating crucial genes in the red light signaling cascade, and (2) the mechanism underlying the dependence of red light signal.

## RESULTS

### Enhanced red light signal increases *F. verticillioides* resistance in maize

**Figure 1:**
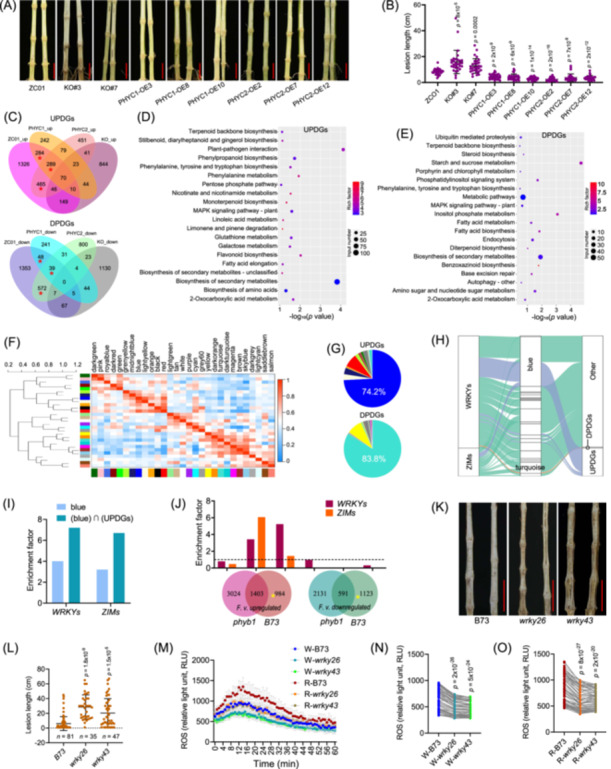
Importance of PHYC‐dependent signaling and WRKY transcription factors in defense against *F. verticillioides*. (A, B) High‐density planting maize (120,000 plants per hectare) was exposed to *F. verticillioides* in the field. KO#3 and KO#7 are two lines of *phyc1 phyc2* double mutants in the ZC01 genetic background. PHYC1‐OE3, PHYC1‐OE8, and PHYC1‐OE10 are three lines of *PHYC1*‐overexpressing transgenic plants also in ZC01 background. Similarly, PHYC2‐OE2, PHYC2‐OE7, and PHYC2‐OE12 are three lines of *PHYC2*‐overexpressing transgenic plants in the same background. ZC01 represents the segregated wild type from the transgenic plants. The data are represented as the mean ± standard deviation (SD) (*n* = 30). The experiment was performed twice, with results of one representative experiment shown. Scale bar = 5 cm. (C) *F. verticillioides* up‐ and downregulated genes in stalks of different samples were used to plot the Venn diagrams. The gene sets marked with red asterisks can be called *F. verticillioides* up‐regulated PHYC‐dependent genes (UPDGs) and *F. verticillioides* down‐regulated PHYC‐dependent genes (DPDGs). (D, E) Kyoto Encyclopedia of Genes and Genomes (KEGG) enrichment analysis of UPDGs and DPDGs, respectively. (F) Based on transcription levels, all genes detected from 36 samples were grouped into 29 gene modules using Weighted Correlation Network Analysis (WGCNA). (G) UPDGs and DPDGs were analyzed for their distribution in 29 gene modules, respectively. Notably, 74.2% of UPDGs and 83.8% of DPDGs are enriched in the blue and turquoise modules, respectively. (H) A Sankey diagram illustrates the distribution of WRKYs, ZIMs, and UPDGs within the blue module. (I) The enrichment factors of the transcription factors WRKY and ZIM in the blue module, as well as the intersection of the blue module and UPDGs, are presented. (J) Venn diagrams were generated using *F. verticillioides* up‐ and downregulated genes from the stalks of B73 and the *phyb1* mutants. The enrichment factors of WRKYs and ZIMs across different gene sets of the Venn diagrams are shown as histograms, with yellow asterisks denoting the *F. verticillioides* upregulated and downregulated PHYB1‐dependent genes. The dotted line indicates that the ordinate value is one. (K, L) Normal‐density planting B73 and *wrky* mutants (52,500 plants per hectare) were exposed to *F. verticillioides* in the field. The data are represented as the mean ± SD. The experiment was performed twice, with results of one representative experiment shown. Scale bar = 10 cm. (M‒O) Maize seedlings were pretreated with red or white light (1200 lux) for 2 days, and then the detached leaves were used for the dynamic monitoring of chitin‐elicited reactive oxygen species. The amount of ROS is represented by relative light unit (RLU). The data are represented as the mean ± SD (*n* = 8). The experiment was performed three times, with the results of one representative experiment shown. The means of each time point were analyzed using a two‐tailed paired Student's *t*‐test to determine the statistical significance of the observed differences in comparison to the wild‐type B73. Statistical analysis was performed using unpaired Student's *t*‐test (two tailed) in (B, K, N). ROS, reactive oxygen species.

PHYB and PHYC are the primary red light receptors responsible for perceiving changes of R:FR [9, 10]. The response to red light was enhanced in plants overexpressing *ZmPHYC1*, *ZmPHYC2*, or *ZmPHYB1*, which is beneficial for high‐density plantings [9, 11, 12]. The *Zmphyb1* and *Zmphyc1 Zmphyc2* double mutants displayed significantly greater susceptibility to *F. verticillioides* than the wild‐type plants (Figure S3). Conversely, overexpression of *ZmPHYC1* or *ZmPHYC2* significantly increased resistance of maize to *F. verticillioides* (Figure S3). The effects of *PHYCs* and *PHYB1* on *F. verticillioides* resistance were continued observed in plants grown under high‐density conditions in the field (Figure 1A,B, and Figure S3I,J).

Next, differences in plant responses to *F. verticillioides* invasion following *PHYC* overexpression or knockout were elucidated using transcriptomic and metabolomic analysis. In the wild‐type plants, 2091 differentially expressed genes (DEGs) were downregulated, and 2639 were upregulated by *F. verticillioides* invasion (Figure 1C, Figure S4A). In plants overexpressing *PHYC1* or *PHYC2*, 62.7% and 59.4% of the upregulated genes, and 22.3% and 41.7% of the downregulated genes, overlapped with those in the wild type (Figure S4A). Conversely, only 22.4% of the upregulated genes and 6.2% of the downregulated genes overlapped between the knockout mutants and the wild‐type (Figure S4A), suggesting that the majority of *F. verticillioides* invasion‐responsive genes were PHYC‐dependent. DEGs and differentially expressed metabolites (DEMs) induced by *F. verticillioides* invasion in wild‐type and overexpression plants, which were absent in knockout mutants, are likely PHYC‐dependent for defense responses. These genes and metabolites are, therefore, referred to as PHYC‐dependent DEGs (PDGs) and DEMs (PDMs). In total, 659 and 1038 PDGs were downregulated (DPDGs) and upregulated (UPDGs) by *F. verticillioides* invasion, respectively (Figure 1C, Tables S1 and S2).

UPDGs were mainly enriched in pathways related to plant‐pathogen interactions, phenylalanine metabolism, flavonoid biosynthesis, and phenylpropanoid biosynthesis, all of which are predominantly linked to defense (Figure 1D). In contrast, DPDGs were enriched in starch and sucrose metabolism, inositol phosphate metabolism, phosphatidylinositol signaling, and fatty acid biosynthesis, all of which are primarily associated with growth (Figure 1E). These results indicated that PHYC‐dependent signaling cascade is essential for defense against *F. verticillioides*.

### WRKY transcription factors may be critical for PHYC/B‐dependent defense responses

Weighted correlation network analysis identified two outstanding gene modules, the blue and turquoise modules, as 74.2% of UPDGs and 83.8% of DPDGs were enriched in these two modules, respectively (Figure 1F,G and Table S3). These results suggested that these two modules play an important role in PHYC‐dependent response to *F. verticillioides* invasion. The top 20 genes with the highest correlation with eigengenes in the two modules were extracted, 19 of the top 20 genes in the blue module belonged to UPDGs (Figure S4B), and 13 of the top 20 genes in the turquoise module belonged to DPDGs (Figure S4C). These genes, such as phospholipid transport ATPase, chitinase, DAG PE‐binding protein, and phosphatidylinositol kinase are important for defense responses. Moreover, the transcription factor *WRKY*s and *ZIM*s were enriched in the blue module (4 fold for *WRKY*s and 3.2 fold for *ZIM*s) and were further enriched in the intersection of UPDGs and the blue module (7.2 fold for *WRKY*s and 6.7 fold for *ZIM*s) (Figure 1H,I Figure S4D,E). The transcriptional responses of *phyb1* mutants and the wild‐type B73 to *F. verticillioides* indicated that *WRKY* transcription factors were enriched in the gene group that is induced by *F. verticillioides* and PHYB1‐dependent (Figure 1J). In contrast, *ZIM* transcription factors were not enriched in this group (Figure 1J). These findings suggest that *WRKY* transcription factors play a general critical role in PHYC‐ and PHYB‐dependent defense mechanisms.

To confirm the role of *WRKY*s enriched in the intersection of UPDGs and the blue module (Figure 1I and Figure S4D), stop codon‐gained mutants of *WRKY43*, *WRKY57*, *WRKY26*, *WRKY125*, and *WRKY48* were purchased from maizeEMSDB (http://maizeems.qlnu.edu.cn/), whereas only *wrky43* and *wrky26* mutants were successfully identified. Both *wrky43* and *wrky26* mutants exhibited consistently greater susceptibility to stalk rot caused by *F. verticillioides* compared to the wild type across various environments (Figure 1K,L). Moreover, the chitin‐induced reactive oxygen species level was lower in *wrky43* and *wrky26* than in the wild type, and the difference increased after the seedlings were pretreated with red light (Figure 1M‒O).

### Thiamine and histamine are key metabolites that improve resistance to *F. verticillioides*

**Figure 2:**
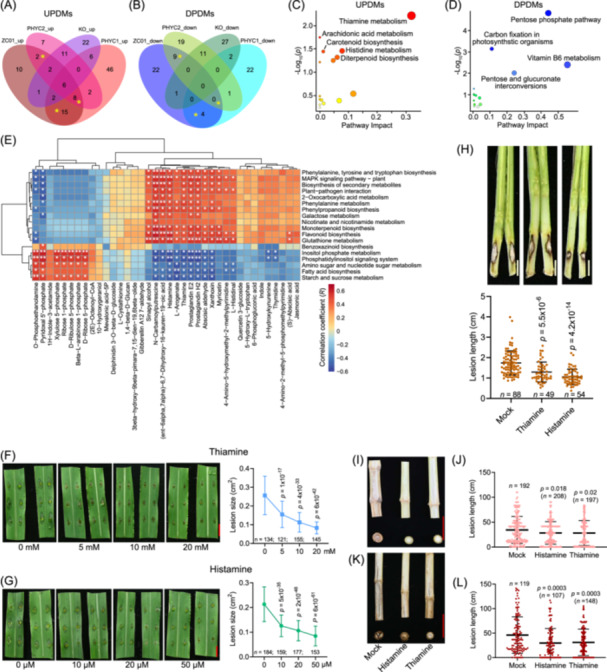
Histamine and thiamine enhance resistance to *F. verticillioides* in maize. (A, B) Venn diagrams illustrate up‐ and downregulated metabolites in stalks of different samples after exposure to *F. verticillioides*. Yellow asterisks‐indicated gene sets are *F. verticillioides* up‐regulated PHYC‐dependent metabolites (UPDMs) and *F. verticillioides* down‐regulated PHYC‐dependent metabolites (DPDMs). (C, D) Pathway enrichment analysis of UPDMs and DPDMs, respectively. (E) Correlation cluster analysis was performed using the expression levels of Kyoto Encyclopedia of Genes and Genomes (KEGG) terms significantly enriched in (D, E), along with the metabolite levels of UPDMs and DPDMs. A log2 conversion and Z‐score on the Reads Per Kilobase per Million mapped reads (RPKM) value of the genes in the target KEGG terms were performed, with the average reflecting the expression levels of the corresponding KEGG terms. Next, a log10 conversion and *Z*‐score on the metabolite abundance data were performed to represent the metabolite levels. A white asterisk indicates significant relevance. *p < 0.01, **p < 0.001. (F, G) Detached leaves were pretreated with thiamine or histamine at different concentrations for 6 h, followed by exposure to *F. verticillioides* for two days; lesion sizes were recorded. The data are represented as the mean ± SD, from three biological replicates. (H) In greenhouse trials, 100 mL of metabolites was applied at a concentration of 20 mM for thiamine and 50 μM for histamine to the roots one day before inoculation with *F. verticillioides*, and stalk lesion lengths were investigated after seven days. The data are represented as the mean ± SD, from three biological replicates. (I‒L) In field trials, the volume of metabolites applied was increased to 500 mL. Stalk rot phenotype was evaluated when the seeds were fully mature. Plants in (I, J) were grown under normal‐density conditions (52,500 plants per hectare), while those in (K, L) were under high‐density conditions (120,000 plants per hectare). The data were calculated from Table S6. To combine data from different environments, we used mock groups to normalize the data from different environments. Scale bars represent 1 cm in (F–H) and 5 cm in (I, K).

In total, 25 PDMs were upregulated (UPDMs), and 13 were downregulated (DPDMs) due to the invasion of *F. verticillioides*, according to metabolomic analysis (Figure 2A,B, Tables S4, S5). UPDMs were mainly enriched in thiamine metabolism, histidine metabolism, and diterpenoid biosynthesis (Figure 2C and Table S4). DPDMs were mainly enriched in the pentose phosphate pathway, vitamin B6 metabolism, carbon fixation, and pentose and glucuronate interconversions (Figure 2D and Table S5). This aligns with the Kyoto Encyclopedia of Genes and Genomes (KEGG) enrichment analysis of UPDGs and DPDGs, in which carbohydrates metabolism pathway and terpenoids biosynthesis pathway were also enriched, respectively.

Correlation clustering analysis showed that several PDMs, such as thiamine, histamine, N‐carbamoylputrescine, prostaglandin, O‐phosphoethanolamine, and pyridoxal 5'‐phosphate, were significantly relevant to some of the enriched KEGG pathways of PDGs, and were clearly clustered into two groups (Figure 2E). The contents of thiamine, histamine, N‐carbamoylputrescine, and prostaglandin were positively relevant to defense‐related KEGG pathways and negatively relevant to growth‐related KEGG pathways; however, the relevance between KEGG pathways and the contents of O‐phosphoethanolamine and pyridoxal 5'‐phosphate showed a completely opposite pattern (Figure 2E). Thiamine enhances plant immune responses to pathogens across various trophic levels [13]. N‐carbamoylputrescine, a precursor of putrescine, plays multiple roles in plant development and stress alleviation [14]. O‐phosphoethanolamine is crucial for phospholipid biosynthesis, acting as a signaling molecule for internal and external stimuli, while phosphatidylinositol promotes *Magnaporthe oryzae* invasion in rice [15]. Pyridoxal 5'‐phosphate is a vital cofactor for metabolic enzymes and stress adaptation [16]. Histamine is a well‐known bioamine with multiple physiological activities particularly mediating the deleterious effects accompanying allergic reactions [17]. In plants, a process resembling an allergic reaction known as the hypersensitive response (HR) is commonly associated with defense mechanisms and is characterized by bursts of ROS. Prostaglandins, crucial signaling molecules in humans, share a similar structure with phytoprostanes and jasmonic acid, both of which are vital signaling molecules in plants, especially in defense responses [18]. However, the functions of histamine and prostaglandins in plants are poorly understood.

Effects of exogenous applications of thiamine, histamine, N‐carbamoylputrescine, prostaglandin E2, O‐phosphoethanolamine, and phosphatidylinositol on maize's resistance to *F. verticillioides* were investigated. Thiamine and histamine consistently enhanced the resistance to *F. verticillioides*, whereas O‐phosphoethanolamine and phosphatidylinositol inhibited this response (Figure 2F,G, and Figure S5). Prostaglandin E2 showed no significant promoting or inhibitory effects, whereas N‐carbamoylputrescine exhibited a more complex influence (Figure S5A‒H). We speculated that histamine and thiamine pretreatment could enhance the burst of ROS under chitin excitation, similarly to red light pretreatment, while O‐phosphoethanolamine and Phosphatidylinositol might exhibit the opposite effect. Unexpectedly, chitin‐induced ROS levels remained unaffected by histamine, O‐phosphoethanolamine, and Phosphatidylinositol, but were suppressed by thiamine treatment (Figure S5I,J). These results indicated that the mechanism by which histamine contributes to defense responses in maize differs from its role in allergic reactions in animals. The effects of thiamine and histamine were further validated by observing the symptoms of stalk rot in greenhouse and field conditions (Figure 2H‒L). Moreover, the disease resistance conferred by thiamine and histamine was found to be more pronounced and stable under high‐density planting conditions than under normal‐density planting conditions (Figure 2I‒L and Table S6).

## DISCUSSION

An ideal plant architecture is beneficial for dense planting in maize. Therefore, harnessing genes that regulate leaf angle to achieve an optimal plant architecture is a key strategy for promoting dense maize plantings [8]. Additionally, manipulating crucial genes in the light signaling cascade to attenuate shade avoidance responses (SARs) represents another strategy for achieving dense planting. Several studies have shown that overexpression of *PHYB*, *PHYC*, or hyperactive variations of *PHYB* can reduce plant and ear heights, conferring attenuated SARs under dense planting conditions and is potential for breeding high‐density tolerant maize cultivars [9, 11, 12]. Here, we found that enhanced *PHYC*‐dependent red light signaling benefits pathogen resistance, particularly under high‐density planting conditions. Therefore, enhancing red light signals may be an ideal strategy for sustainable agriculture, improving disease management and yield stability in high‐density conditions.

We identified two defense‐related gene networks and several metabolites that are dependent on *PHYC*s, involving key contributors such as WRKY transcription factors and metabolites like histamine and thiamine. Although histamine plays a significant role in allergic reactions in animal tissues [17], and both allergic reactions in animals and hypersensitivity responses in plants are intrinsic immune responses, there have been no reports to date demonstrating that histamine is involved in plant immune responses. Thus, it may be the most interesting finding in this study. Notably, histamine pretreatment did not influence the ROS burst as the *WRKY26* and *WRKY43* genes did under chitin induction, indicating that the mechanism by which histamine contributes to defense responses in maize differs from that of *WRKY26* and *WRKY43*. Histamine has been reported to act as a signaling molecule in bacteria, triggering chemoattraction [19]. Thus, it may facilitate the recruitment of beneficial microorganisms around the roots, aiding maize combating *F. verticillioides* in the field. Additionally, histamine is structurally similar to dopamine, and perhaps its mechanism of enhancing plant disease resistance is also similar to that of dopamine [20]. Histamine is a cheap natural compound that has a lower environmental impact than usual fungicides. Therefore, it may have the potential to be developed into a new type of environmentally friendly chemical control agent for plant protection.

In animals, the various biological activities of histamine were attributed to G‐protein‐coupled receptors. However, the role of histamine in plants has not garnered significant research attention, leaving the existence of histamine receptors in plants unclear. One limitation of our study is that we did not establish a direct connection between red light signaling and histamine synthesis. Additionally, the mechanisms by which histamine enhances maize resistance to *F. verticillioides* remain poorly understood. Nonetheless, it is important to recognize that histamine may play a role in regulating plant immunity. Unraveling the mechanism by which histamine regulates plant immunity would be an interesting avenue for future research. As we deepen our understanding of histamine's role in plants, the question of whether histamine receptors exist will also become an intriguing scientific inquiry.

## CONCLUSION

This study highlights the potential of enhancing red light signaling and histamine in improving maize resistance to pathogens under dense planting conditions. The role of histamine in plant immunity remains largely unexplored, presenting opportunities for future research on its mechanisms and potential as an environmentally friendly biocontrol agent.

## METHODS

Detailed information about the methods and materials is available in the Supplementary Information.

## AUTHOR CONTRIBUTIONS

**Xuanjun Feng**: Conceptualization; methodology; investigation; formal analysis; supervision; funding acquisition; visualization; resources; writing—original draft; writing—review and editing. **Dan Zheng**: Methodology; data curation; investigation; validation; formal analysis; visualization; writing—original draft; writing—review and editing. **Weixiao Zhang**: Methodology; data curation; investigation; validation; formal analysis; visualization; writing—original draft; writing—review and editing. **Huihui Xiao**: Data curation; investigation; validation; writing—review and editing. **Huarui Guan**: Data curation; investigation; validation; writing—review & editing. **Hao Xiong**: Data curation; Investigation; validation; writing—review and editing. **Li Jia**: Validation; data curation; investigation; writing—review and editing. **Xuemei Zhang**: Data curation; investigation; project administration; writing—review and editing. **Wenming Wang**: Writing—review and editing. **Haiyang Wang**: Writing—review and editing; resources. **Yanli Lu**: Funding acquisition; project administration; resources; supervision; writing—original draft; writing—review & editing.

## CONFLICT OF INTEREST STATEMENT

The authors declare no conflicts of interest.

## ETHICS STATEMENT

No animals or humans were involved in this study.

## Supporting information


**Figure S1.** Red light has the best effect on promoting *F. verticillioides* resistance in maize.
**Figure S2.** Red light supplementation enhances maize resistance to *F. verticillioides*.
**Figure S3.** *ZmPHYC*s and *ZmPHYB1* genes are important for resistance to *F. verticillioides* invasion in maize.
**Figure S4.** WRKY and ZIM transcription factors are enriched in the PHYC‐dependent defense gene module.
**Figure S5.** O‐phosphoethanolamine and phosphatidylinositol decrease maize resistance to *F. verticillioides*.
**Figure S6.** Framework of this study.
**Figure S7.** Clustering heatmaps of the transcriptomic data of PHYC1 overexpressing plants, PHYC2 overexpressing plants, *phyc1 phyc2* double mutants, and the wild‐type ZC01.
**Figure S8.** Clustering heatmaps of the transcriptomic data of *phyb1* and the wild‐type B73.
**Figure S9.** Clustering heatmaps of the metabolomic data of PHYC1 overexpressing plants, PHYC2 overexpressing plants, *phyc1 phyc2* double mutants, and the wild‐type ZC01.
**Figure S10.** Ten genes with different expression levels were selected to validate the RNA‐seq results.

**Table S1.** *F. verticillioides* upregulated and PHYC‐dependent differentially expressed genes (UPDGs) with average RPKM for each sample.
**Table S2.** *F. verticillioides* downregulated and PHYC‐dependent differentially expressed genes (DPDGs) with average RPKM for each sample.
**Table S3.** Gene list in different modules of WGCNA result.
**Table S4.** *F. verticillioides* upregulated and PHYC‐dependent differentially expressed metabolites (UPDMs) with average relative abundance for each sample.
**Table S5.** *F. verticillioides* downregulated and PHYC‐dependent differentially expressed metabolites (DPDMs) with average relative abundance for each sample.
**Table S6.** Lesion length of stalk rot under different metabolites treatment.
**Table S7.** Sequencing data and QC summary table for *PHYC*.
**Table S8.** Sequencing data and QC summary table for *phyb1*.
**Table S9.** Primers utilized in this study.

## Data Availability

The data that supports the findings of this study are available in the supplementary material of this article. RNA‐seq and metabolome data can be accessed through the National Center for Biotechnology Information (NCBI) (https://www.ncbi.nlm.nih.gov/sra/?term=PRJNA1149317) and the China National Center for Bioinformation (CNCB) (https://ngdc.cncb.ac.cn/bioproject/browse/PRJCA029223). All other data are available in the main text or supporting information. Supplementary materials (results, methods, figures, tables, graphical abstract, slides, videos, Chinese translated version, and update materials) may be found in the online DOI or iMeta Science (http://www.imeta.science/).
