# Supplementary material for: Histamine: A key compound in red light‐enhanced Fusarium verticillioides resistance in maize
Source: Imeta. 2025 Apr 7;4(2):e70020. doi: 10.1002/imt2.70020 (PMC11995177; doi:10.1002/imt2.70020)
Supplement: Supplementary file 1 — Figure S1. Red light has the best effect on promoting F. verticillioides resistance in maize. Figure S2. Red light supplementation enhances maize resistance to F. verticillioides. Figure S3. ZmPHYCs and ZmPHYB1 genes are important for resistance to F. verticillioides invasion in maize. Figure S4. WRKY and ZIM transcription factors are enriched in the PHYC‐dependent defense gene module. Figure S5. O‐phosphoethanolamine and phosphatidylinositol decrease maize resistance to F. verticillioides. Figure S6. Framework of this study. Figure S7. Clustering heatmaps of the transcriptomic data of PHYC1 overexpressing plants, PHYC2 overexpressing plants, phyc1 phyc2 double mutants, and the wild‐type ZC01. Figure S8. Clustering heatmaps of the transcriptomic data of phyb1 and the wild‐type B73. Figure S9. Clustering heatmaps of the metabolomic data of PHYC1 overexpressing plants, PHYC2 overexpressing plants, phyc1 phyc2 double mutants, and the wild‐type ZC01. Figure S10. Ten genes with different expression levels were selected to validate the RNA‐seq results. [file IMT2-4-e70020-s001.docx]

**Histamine: a key compound in red light-enhanced *Fusarium verticillioides* resistance in maize**

**Running title:** Histamine boosts plant disease resistance

Xuanjun Feng^1,2#^, Dan Zheng^2#^, Weixiao Zhang^2#^, Huihui Xiao^1^, Huarui Guan^2^, Hao Xiong^2^, Li Jia^2^, Xuemei Zhang^2^, Wenming Wang^1^, Haiyang Wang^3^, Yanli Lu^1,2^*

^1^State Key Laboratory of Crop Gene Exploration and Utilization in Southwest China, Sichuan Agricultural University, Wenjiang, 611130, China.

^2^Maize Research Institute, Sichuan Agricultural University, Wenjiang, 611130, China.

^3^State Key Laboratory for Conservation and Utilization of Subtropical Agro-Bioresources, South China Agricultural University, Guangzhou, 510642, China.

^#^These authors contribute equally to this work: Xuanjun Feng, Weixiao Zhang, Dan Zheng.

*Correspondence: [luyanli@sicau.edu.cn](mailto:luyanli@sicau.edu.cn) (Yanli Lu)

**Supplementary Results**

**Red light enhances *F. verticillioides* resistance in maize**

High-density conditions caused typical shade avoidance syndromes with internode elongation and stalk diameter reduction (Figure S1A‒C). The stalk rot caused by *F. verticillioides* was dramatically more severe in the high-density condition than in the normal-density condition (Figure S1A,D,E). The mutual shading of the leaves significantly reduces the ratios of red to far-red (R:FR), blue to far-red (B:FR), and ultraviolet to far-red (UV:FR) light, as well as the overall light intensity under the canopy (Figure S1F). Although red light has been widely reported to promote plant defense, blue and ultraviolet have also been reported to promote plant defense against some diseases [1‒4]. To investigate the effects of different light on *F. verticillioides* resistance, maize seedlings were primed under low light intensity conditions (80 lux) with red (630 nm), far-red (730 nm), blue (460 nm), white, and UV light (313 nm, UV intensity of 15 lux, and 65 lux white light was supplemented) for two days, and then leaves or seedling stalks were exposed to *F. verticillioides* XY-1 under the original light conditions*.* Red light showed the most exciting promoting effect on *F. verticillioides* resistance in both leaf and stalk (Figure S1G‒L). The similar results for *Magnaporthe oryzae* resistance in rice implied that red light may have a universal effect in defense against pathogen invasion (Figure S1M,N). Unexpectedly, far-red light also has a stimulating effect on *F. verticillioides* resistance although with less amplitude. In addition, red light has an inhibitory effect on *F. verticillioides* directly during the spore germination stage (Figure S1O‒R). Therefore, different light-primed leaves were incubated in the dark after inoculation with *F. verticillioides*. Results showed that only red light primed leaves displayed a weaker symptom and lower colonization of spores than that of the white light primed leaves (Figure S1S‒V).

The effects of supplementing different light qualities were further investigated. Supplementation of red or far-red light dramatically improved maize resistance to *F. verticillioides* (Figure S2A‒G). Since the light intensity under the maize canopy is about 2400 lux in natural conditions (Figure S1F), the effects of adding red or far-red light were further investigated at an intensity of 2500 lux (supplementing 500 lux red light to 2000 lux white light). Red light supplementation continued to improve *F. verticillioides* resistance, whereas far-red light showed no significant effect (Figure S2H). The effect of red light was further investigated by supplementing 500 lux red light alongside 4000 lux or 49,500 lux of white light. The inclusion of 500 lux red light continued to promote *F. verticillioides* resistance under 4500 lux conditions, but this effect was not observed at the much higher intensity of 50,000 lux (Figure S2I‒K). These results imply that the enhancement of red light signal under the canopy of maize may potentially improve its disease resistance in the field. Reactive oxygen species (ROS) burst is a common basic defensive weapon against broad-spectrum pathogen invasion. ROS burst in response to chitin, a common pathogen-associated molecular pattern (PAMP) of fungi, was compared between red light, white light and dark primed seedlings. Chitin-induced ROS were highest in red light primed plants and lowest in the dark primed plants (Figure S2L,M). The results indicate that enhancing red light signals could effectively manage diseases, particularly in dense plantings.

**Materials and Methods**

**Research framework**

The study is organized into four main steps (Figure S6). First, we examined how different light qualities affect maize’s resistance to *F. verticillioides*. Our findings reveal that red light is the most effective wavelength for enhancing this resistance. Additionally, providing red light at an intensity comparable to that found under an in-field canopy can significantly improve maize’s defense against this pathogen. Second, we investigated the effects of manipulating red light receptors on maize’s resistance to *F. verticillioides*. Our results indicate that boosting red light signaling through receptor manipulation significantly enhances resistance to the pathogen. Third, we employed a combined transcriptome and metabolome approach to elucidate the mechanisms underlying PHYC-dependent resistance to *F. verticillioides*. Finally, we identified several genes and metabolites dependent on *PHYC*s that are involved in resistance to *F. verticillioides*, including key transcription factors such as *WRKY26* and *WRKY43*, as well as important metabolites like histamine and thiamine.

**Plant materials**

*PHYC*-overexpressing plants and knockout mutants were kindly provided by Professor Haiyang Wang from South China Agricultural University [5]. Stop codon-gain mutants of *phyb1* (EMS3-0058f4), *wrky43* (EMS4-1087bf), *wrky57* (EMS4-1b322e), *wrky26* (EMS4-0a4bda), *wrky125* (EMS4-1b165a), and *wrky48* (EMS4-077828) were purchased from maizeEMSDB (<http://maizeems.qlnu.edu.cn/>). Before the phenotypic analysis, the *phyb1* and *wrky* mutants underwent two rounds of backcrossing with wild-type B73. The wild-type maize used in this study was derived from the segregation of transgenic or backbred progenies. KO#3 and KO#7 are two lines of *phyc1 phyc2* double mutants in the ZC01 genetic background. C1#OE3, C1#OE8, and C1#OE10 are three lines of *PHYC1*-overexpressing transgenic plants also in ZC01 background. Similarly, C2#OE2, C2#OE7, and C2#OE12 are three lines of *PHYC2*-overexpressing transgenic plants in the same background. ZC01 represents the segregated wild type from the transgenic plants. Since the genetic background of the *phyb1* mutant is B73, B73 was used as the wild-type control in related experiments. The Kitaake rice cultivar was used to test *Magnaporthe oryzae* resistance.

**Treatment under different light conditions**

Thirty-day-old maize or rice seedlings were pretreated under low-light intensity conditions (80 lux) with red (630 nm), far-red (730 nm), blue (460 nm), white, or UV light (313 nm, UV intensity of 15 lux, supplemented with 65 lux white light) for two days. For leaf resistance assessment, detached leaves were exposed to *F. verticillioides* XY-1 under the original light conditions*.* A middle section (approximately 6 cm) of the third and fourth healthy leaves was used for inoculation. The pipette tip was gently pressed and rotated on the adaxial surface of the leaf to create six lesion points, to which 2 μL of spore droplets (5 × 10^6^ spores/mL) were added. The leaves were subsequently floated in distilled water containing 1 mg/L of 6-benzylaminopurine. To exclude the effect of direct light on *F. verticillioides*, detached leaves were kept in the dark following inoculation with the spore solution. For stalk resistance, a 2 mm needle was used to inject 3 μL of spore solution into the stalk, with the injection site sealed with paraffin. The seedlings were then transferred to normal light conditions in a greenhouse for approximately seven days.

To investigate the effect of different wavelengths of light supplementation, seedlings were pretreated under an intensity of 200 lux (comprising 80 lux red, far-red, and blue light, along with 15 lux UV light added to white light) conditions for two days, after which detached leaves or seedlings were exposed to *F. verticillioides* XY-1 under the original light conditions. The seedling lodging rate was assessed by irrigating the roots with spore solution. The lodging rate and lesion length of the stalks were measured approximately seven days post-inoculation. To further examine the effects of red and far-red light, 500 lux of either red or far-red light was added to a higher white light intensity to simulate natural conditions. Lesion size and length were quantified using the ImageJ software.

**Microbial strains and culture conditions**

*F. verticillioides* XY-1 was isolated from infected maize ears in the field using a single-spore isolation method [6]. The corn sand was soaked in hot water (80 ℃) for two hours, after which the filtrate was autoclaved and used for the cultivation of XY-1. Following growth for 3‒5 days at 28 ℃ in the dark, the spore concentration exceeded 5 × 10^6^ spores/mL, making it suitable for inoculation. The corn kernels were soaked for 12 h, cooked, cooled, and then mixed with the spore solution. This mixture was cultured for approximately one week, with frequent stirring during the incubation period before field inoculation.

**Detection of ROS burst**

ROS levels were measured following elicitor treatment. Briefly, a 3 mm diameter leaf disc was placed into the wells of a 96-well white microplate. The disc was rinsed overnight with distilled water at 25 ℃. After removing the distilled water, 200 μL of fresh distilled water was added for a second wash. Once this was removed, 200 μL of a luminol mixture (200 μM luminol and 20 μg/mL peroxidase) was added, and the mixture was incubated at 30 ℃ for 30 min. Subsequently, 10 μL of N,N',N,N''',N'''',N'''''-hexaacetyl chitohexaose (Solarbio, SN8990, China) at 100 μg/mL was quickly added to each well. Signal acquisition took place at 30 ℃ using a SpectraMax L microplate reader (Molecular Devices, USA) over one hour, with measurements taken every minute. Eight replicates were used for each treatment and ROS levels at each time point were expressed in relative luminescence units (RLU).

**Observation of spore germination and colonization on the leaf surface**

To investigate the effect of different wavelengths of light on the growth of XY-1, 4 μL of spore droplets were placed on a hydrophobic slide, covered with a coverslip, and sealed with petroleum jelly. The slides were exposed to various light conditions for 4, 8, and 12 h, and the spore germination rate was assessed through microscopic imaging at each time point using a ×20 objective. The center and four corners of each slide were examined, with 30 slides observed per treatment across 5 replicates. To observe colony growth at different wavelengths, the spore solution was diluted to approximately 1000 per milliliter. Then, 10 μL of spore solution was added to the center of each potato dextrose agar solid Petri dish, which was subsequently incubated under different light conditions. After four days, the colonies were photographed, and the colony diameter was measured using ImageJ. Each treatment was repeated 10 times, and each colony was measured twice in a criss-cross manner.

To examine the spore colonization on the leaf surface, undamaged leaves were floated on the spore solution and kept in the dark for 16 h. The leaves were then treated with ethanol at 70 ℃ to remove chlorophyll. Afterward, they were stained with an acid fuchsin solution (10 mL of phenol, 10 mL of glycerin, 10 mL of lactic acid, and 3 mg of acidic fuchsin) for 48 h, rinsed twice with clean water, and subsequently observed under a microscope. To determine the spore colonization rate, six regions were uniformly selected from the center of each leaf, and images were acquired from six leaves using a ×20 objective.

**RNA-seq analysis**

Thirty-day-old maize seedlings (#OE3 of *PHYC1* overexpressing line, #OE12 of *PHYC2* overexpressing line, *phyc1 phyc2* double mutant KO7, and the segregated wild-type ZC01; *phyb1* mutants and the segregated wild-type B73) were pretreated under 1200 lux of red light for two days. The seedling stalks were then exposed to either *F. verticillioides* XY-1 or sterile water (mock) under the original light conditions. After 10 and 24 h, stalk segments approximately 1 cm long around the inoculation site were collected from 10 plants, ground in liquid nitrogen, and aliquoted into six samples: three for RNA sequencing and three for metabolome analysis.

Strand-specific RNA sequencing and data analysis were conducted by Seqhealth Technology Co., LTD (Wuhan, China). Total RNA was extracted from stalks using TRIzol Reagent (Invitrogen, 15596026, USA). RNA quality was assessed by examining the A260/A280 ratio using a Nanodrop^TM^ OneC spectrophotometer (Thermo Fisher Scientific Inc). RNA Integrity was confirmed through 1.5% agarose gel electrophoresis. Qualified RNAs were quantified using Qubit3.0 with the QubitTM RNA Broad Range Assay kit (Life Technologies, Q10210). Two micrograms of total RNA were utilized for stranded RNA sequencing library preparation, following the manufacturer’s instructions, using the KCTM Stranded mRNA Library Prep Kit for Illumina (DR08402, Wuhan Seqhealth Co., Ltd. China). PCR products corresponding to 200‒500 bp were enriched, quantified, and sequenced on a DNBSEQ-T7 sequencer (MGI Tech Co., Ltd., China) using the PE150 model.

Raw sequencing data were initially filtered using Trimmomatic (version 0.36), discarding low-quality reads and trimming those contaminated with adaptor sequences. Information on the sequencing quality is shown in Tables S7 and S8. Clean data were then mapped to the reference genome of *Zea mays* B73 using STRA software (version 2.5.3a) with default parameters. Read counts mapped to the exon regions of each gene were obtained using featureCounts (Subread-1.5.1; Bioconductor), from which the Reads Per Kilobase per Million mapped reads (RPKM) values were calculated. The overall relationship between the samples can be observed in the clustering heat map (Figure S7 and S8). Differential gene expression between the groups was determined using the edgeR package (version 3.12.1), employing a *p*-value cutoff of 0.05 and a fold-change cutoff of 2 for statistical significance. Genes that were differentially expressed at either 10 or 24 h post-infection compared to the mock were considered *F. verticillioides* induced DEGs.

Gene Ontology (GO) and Kyoto Encyclopedia of Genes and Genomes (KEGG) enrichment analysis for differentially expressed genes were performed using KOBAS software (version 2.1.1), with a *p*-value cutoff of 0.05. Alternative splicing events were detected using rMATS (version 3.2.5), applying a false discovery cutoff of 0.05 and an absolute Δψ value of 0.05. Transcripts that differentially expressed at either 10 or 24 h post-infection compared to mock were considered as *F. verticillioides* induced differentially expressed alternatively spliced transcripts (DEAST). We employed Weighted Gene Co-Expression Network Analysis (WGCNA) to construct a weighted correlation network from the gene expression data. All genes detected from 36 samples were used for network construction. First, we calculated the adjacency between genes and constructed a topological overlap matrix. A hierarchical clustering tree was then produced based on the dissimilarity of the topological overlap matrix, and modules were selected using the dynamic three cut method. Finally, similar modules were merged by calculating and clustering the module eigengenes, assigning a distance threshold.

**LC-MS/MS non-targeted metabolome analysis**

Metabolomic profiling was conducted by Shanghai Bioprofile Co., LTD (Shanghai, China) using an UltiMate3000 UHPLC system coupled with a Q-Exactive (Thermo Scientific, San Jose, USA).

The samples were finely ground in liquid nitrogen, and to each sample of equal mass, 0.2 mL of pre-cooled methanol/water solution (7:3, v/v) was added to each sample. The mixture was vortexed, sonicated at low temperature for 30 min, allowed to stand on ice for 10 min, and then centrifuged at 14,000 × g and 4 °C for 20 min. The supernatant was filtered through a 0.22 μm membrane for injection.

For liquid chromatography separation, samples were analyzed using a Poroshell 120 EC-C18 column (Agilent Technologies, USA). The injection volume was 2 μL, with a flow rate of 0.3 mL/min. The mobile phase contained of A: 0.1% formic acid in water and B: 100% acetonitrile. The gradient elution procedure was as follows: buffer B was linearly increased from 2% to 90% over 0 to 35 min, maintained at 90% from 35 to 40 min.

Parameters for the Q-Exactive were set as follows: Level 1 parameters—Analyzer: FTMS; Scan Type: Full; Resolution: 60000; Polarity: +/-; Scan Range: 100–1000 m/z. Level 2 parameters—Analyzer: FTMS; Resolution: 15000; Dynamic Exclusion: 10 s; Charge state: Reject 1; Current Scan Event: Top 15 peaks; Activation Type: HCD; Normalized Collision Energy: 35.

Raw data were converted into the abf format using an ABF converter, followed by peak alignment, retention time correction, and peak area extraction using the MS-DIAL program. Discriminatory metabolites were identified using a statistically significant threshold based on the variable influence on projection (VIP) values from the OPLS-DA model along with two-tailed Student’s *t*-test (*p*-value) of the normalized raw data at the univariate analysis level. The *p*-value was calculated using one-way analysis of variance (ANOVA) for multiple group comparisons. Metabolites with VIP values greater than 1.0, *p*-value less than 0.05, and fold changes greater than 1.5 were considered statistically significant differentially expressed metabolites. The fold change was calculated as the logarithm of the average mass response (area) ratio between two arbitrary classes. The overall relationship between the samples is shown in the clustering heat map (Figure S9). Metabolites that differentially expressed at either 10 or 24 h post-infection compared to mock were considered as DEMs. Metabolite enrichment pathway analysis was performed using MetaboAnalyst 6.0 (<https://dev.metaboanalyst.ca/MetaboAnalyst/upload/PathUploadView.xhtml>).

**Quantitative RT-PCR**

Quantitative RT-PCR was used to investigate the biomass of XY-1 following inoculation and validate the results obtained from RNA sequencing. For biomass determination, inoculated tissues were processed for DNA extraction using a rapid fungi genomic DNA isolation kit (Sangon Biotech, B518229, China). For gene expression analysis, total RNA was extracted from the stalks using a plant total RNA isolation kit (FOREGENE, RE-05014, China). Genomic DNA was removed from the RNA samples using RNase-free DNase I (Trans; GD201-01). RNA concentration was measured using a spectrophotometer (NanoDrop 2000C). First-strand cDNA was synthesized from DNase I-treated RNA using the Prime Script RT reagent kit with gDNA Eraser (Takara; RR047A) from DNase I-treated RNA. Quantitative RT-PCR was performed using SYBR Green Fast qPCR Mix (Abclonal, RM21203) on a Bio-Rad CFX96 machine. The *ZmEF1a* gene served as a reference to normalize the biomass of infected tissues, while 18S rRNA and *ZmEF1a* were used to normalize the expression levels of candidate genes. Ten genes with different expression levels were selected to validate the RNA-seq results. qRT-PCR showed that the RNA-seq results were well reproducible (Figure S10). The primers used for the analyses are listed in Table S9.

**Exogenous metabolite effects**

The effects of thiamine (Solarbio, V8020, China), histamine (Shanghai yuanye, B24434, China), N-carbamoylputrescine (Shanghai yiyao, 6851-51-0(CAS)), prostaglandin E2 (Macklin, C15196906, China), O-phosphoethanolamine (Macklin, O889434, China), and phosphatidylinositol (Macklin, L837155, China) on resistance to *F. verticillioides* were first investigated using detached leaves. The detached leaves were pretreated with different metabolites for 6 h before exposure to *F. verticillioides,* as described previously, or were used for ROS detection following elicitor treatment. Thiamine and histamine were applied to the roots of seedlings in the greenhouse and adult plants in the field. A volume of 100 mL of metabolites was applied at a concentration of 20 mM for thiamine and 50 μM for histamine to the roots one day before inoculation with XY-1 in greenhouse. In the field trials, the volume of the metabolites applied was increased to 500 mL.

**Field trials and phenotyping of stalk rot**

To investigate the severity of stalk rots in the field, the maize seedlings were planted in 2023 at Xishuangbanna (21° 53′ N, 100° 59′ E) and in 2024 at Wenjiang (30° 43′ N, 103° 52' E) and Chongzhou (30° 33′ N, 103° 39′ E). For normal-density planting, 10 seedlings were planted in each row, with a row length of 3 m and a spacing of 0.6 m. For high-density plantings, 20 seedlings were planted per row with a row length of 3 m and a row spacing of 0.5 m. Approximately 60 days after planting, a 10 cm diameter hole was dug near the root, and 50 grams of a corn kernel medium enriched with XY-1 was buried in it to investigate stalk rot. To assess the effect of purchased thiamine and histamine, 500 mL of aqueous metabolites (20 mM for thiamine and 50 μM for histamine) was applied to the roots one day before inoculation with XY-1. The stalk rot phenotype was investigated when seeds were fully mature. Because stalk rot lesions caused by *F. verticillioides* are typically discontinuous, the lesion length in this study refers to the total length of these discontinuous lesions.

**Accession numbers**

*ZmPHYC1* (Zm00001d034038), *ZmPHYC2* (Zm00001d013262), *ZmPHYB1* (Zm00001d028905), *ZmWRKY26* (Zm00001d008794), *ZmWRKY43* (Zm00001d043025), *ZmWRKY57* (Zm00001d038451), *ZmWRKY125* (Zm00001d037607), and *ZmWRKY48* (Zm00001d015515)

**REFERENCES**

1. Galle, Agnes., Zalan. Czekus, Liliana. Toth, Laszlo. Galgoczy, Peter. Poor. 2021. “Pest and disease management by red light.” *Plant Cell and Environment* 44: 3197–3210. <https://doi.org/10.1111/pce.14142>

2. Ballaré, Carlos L. 2014. “Light regulation of plant defense.” *Annual Review of Plant Biology* 65: 335–363. <https://doi.org/10.1146/annurev-arplant-050213-040145>

3. Lauria, Giulia, Ermes Lo Piccolo, Costanza Ceccanti, Lucia Guidi, Rodolfo Bernardi, Fabrizio Araniti, Lorenzo Cotrozzi, et al. 2023. “Supplemental red LED light promotes plant productivity, “photomodulates” fruit quality and increases *Botrytis cinerea* tolerance in strawberry.” *Postharvest Biology and Technology* 198: 112253. <https://doi.org/10.1016/j.postharvbio.2023.112253>

4. Hao, Yuhan, Zexian Zeng, Minhang Yuan, Hui Li, Shisong Guo, Yu Yang, Shushu Jiang, et al. 2024. “The blue-light receptor CRY1 serves as a switch to balance photosynthesis and plant defense.” *Cell Host & Microbe* 33: 1–14. <https://doi.org/10.1016/j.chom.2024.12.003>

5. Li, Quanquan, Guangxia Wu, Yongping Zhao, Baobao Wang, Binbin Zhao, Dexin Kong, Hongbin Wei, Cuixia Chen, Haiyang Wang. 2020. “CRISPR/Cas9-mediated knockout and overexpression studies reveal a role of maize phytochrome C in regulating flowering time and plant height.” *Plant Biotechnology Journal* 18: 2520–2532. <https://doi.org/10.1111/pbi.13429>

6. Xiong, Hao, Xiaobin Xing, Muyuan Liu, Zhaoyu Zhang, Qingjun Wang, Xuemei Zhang, Xiangjian Gou, Yanli Lu, Xuanjun Feng. 2024. “Stalks and roots are the main battlefield for the coevolution between maize and *Fusarium verticillioides*.” *Frontiers in Plant Science* 15: 1461896. <https://doi.org/10.3389/fpls.2024.1461896>


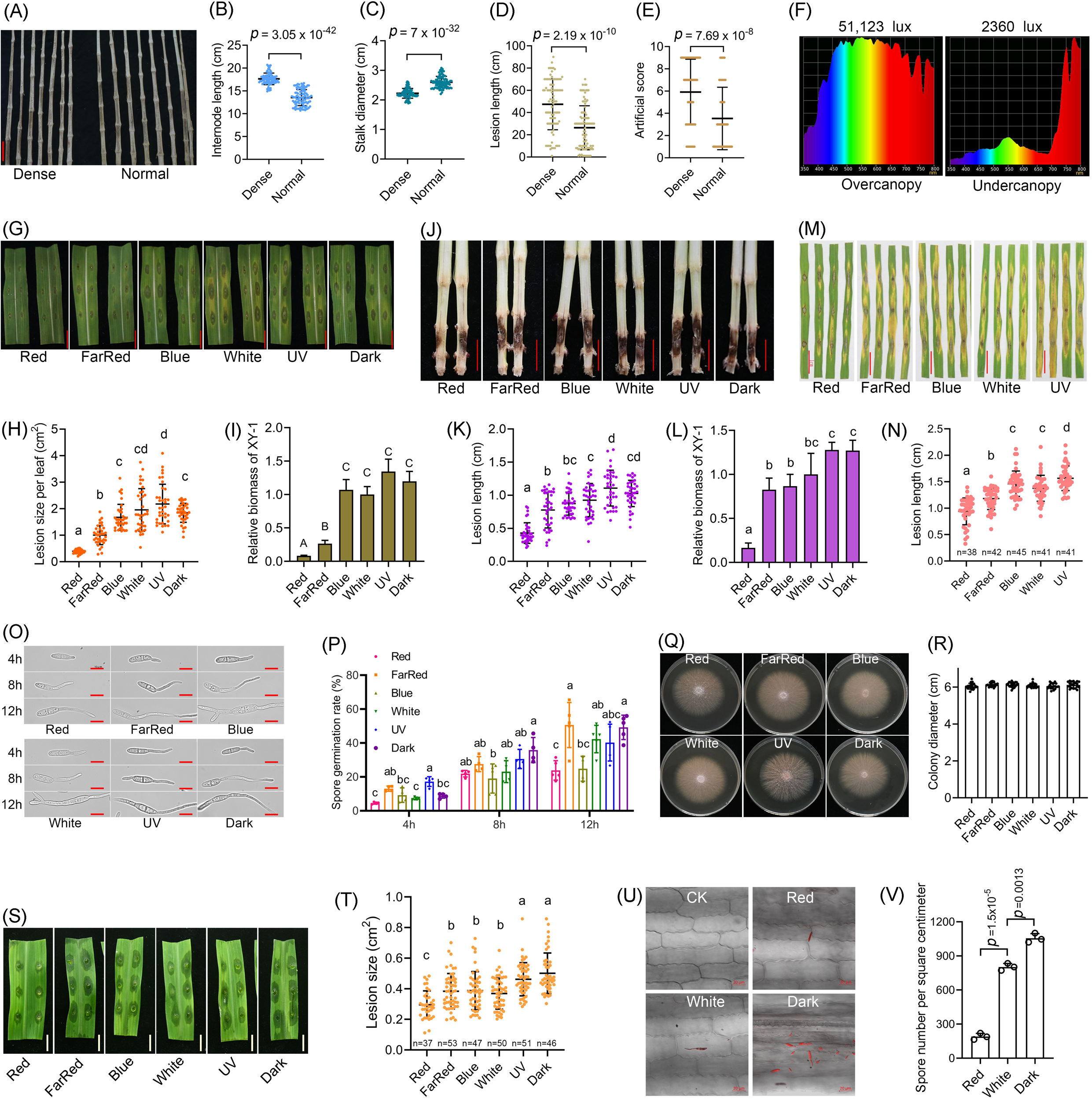


**Figure S1 Red light has the best effect on promoting *F. verticillioides* resistance in maize.** (A) The *F. verticillioides* XY-1 caused a different stalk rot phenotype between high-density and normal-density planting conditions. (B‒E) Statistical analysis of internode length, stalk diameter, lesion length, and severity score of stalk rot. The data are represented as the mean ± SD (*n* = 88 for Dense; *n* = 97 for Normal). Scale bar = 10 cm. (F) Light spectra above and below the canopy of maize under normal-density planting conditions. (G‒I) The maize seedlings were pretreated for two days under different light conditions (80 lux for Red, FarRed, Blue, and White light; 15 lux for UV light). Detached leaves were then exposed to *F. verticillioides* spores for an additional two days under the pretreated light conditions. Lesion size was investigated using ImageJ, and biomass of *F. verticillioides* in the infected leaves was determined through Quantitative RT-PCR. The data are represented as the mean ± SD (*n* = 36), from three biological replicates. (J‒L) The maize seedlings were pretreated as in Figure S1G, and then the live stalks were exposed to *F. verticillioides* spores for seven days under normal greenhouse light conditions (4000 lux). The data are represented as the mean ± SD (*n* = 36), from three biological replicates. (M and N) Rice seedlings were pretreated for two days under different light wavelengths (80 lux intensity for Red, FarRed, Blue, and White light; 15 lux for UV light). Detached leaves were then exposed to *Magnaporthe oryzae* spores for an additional three days under the pretreated light conditions. The data are represented as the mean ± SD, from three replicates. Scale bar = 1 cm. (O and P) Spore germination rate in water under different light wavelengths (80 lux for Red, FarRed, Blue, and White light; 15 lux for UV light). The data are represented as the mean ± SD (*n* = 5), from five replicates. One dot represents the average germination rate of one replicate. Scale bar = 10 μm. (Q and R) Colony sizes of *F. verticillioides* grown on potato dextrose agar under different light wavelengths over four days. The data are represented as the mean ± SD (*n* = 20), from two replicates, with each clone assayed twice. (S and T) Maize seedlings were pretreated for two days with different light wavelengths (80 lux for Red, FarRed, Blue, and White light; 15 lux for UV light), after which detached leaves were exposed to *F. verticillioides* spores for an additional two days in the dark. The data are represented as the mean ± SD. Scale bar = 1 cm. (U and V) Investigation the colonization of *F. verticillioides* on the leaf surface. Maize seedlings were pretreated for two days under red and white light conditions (80 lux), then detached leaves were exposed to *F. verticillioides* spores for 16 h in the dark. Infected leaves were stained with acid fuchsin and the number of spores was counted. CK indicates that the detached leaves were exposed to *F. verticillioides* spores and immediately removed to a non-*F. verticillioides* condition for 16 h in the dark. The data are represented as the mean ± SD (*n* = 3). One dot represents the average germination rate of one replicate. Scale bar = 20 μm.


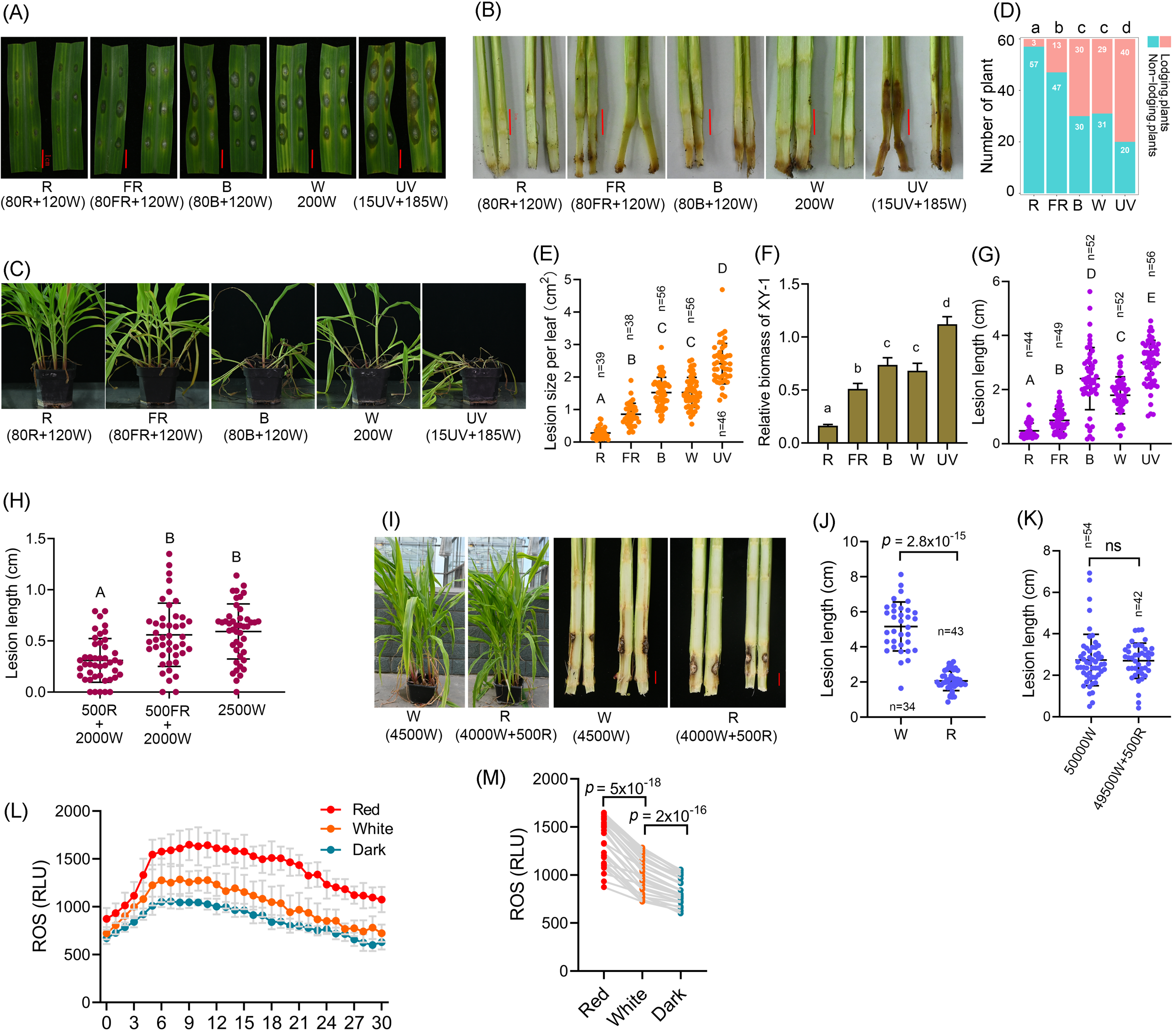


**Figure S2 Red light supplementation enhances maize resistance to *F. verticillioides*.** (A‒G) The maize seedlings were pretreated for two days in five different conditions. The detached leaves were then exposed to *F. verticillioides* spores for two additional days in the pretreated conditions. For stalk rot, the roots were exposed to *F. verticillioides* spores for seven days under the pretreated conditions. The lesion length lodging rate were investigated. The values in the stacked histogram represent the number of corresponding plants. Statistical analysis was performed using GraphPad Prism 8.0.2 with Binomial test under Wilson/Brown method. The different letters assigned to the treatments indicate that the differences between the two are significant (*p* < 0.0332). Data is from three biological replicates (*n* = 60). Scale bar = 1 cm. (H) Maize seedlings grown under three conditions were exposed to *F. verticillioides* spores for approximately seven days, and stalk lesion lengths were investigated. The data are represented as the mean ± SD (*n* = 44), from two replicates. (I‒K) Maize seedlings grown under two conditions were exposed to *F. verticillioides* spores for about seven days, after which stalk lesion lengths were investigated. The data are represented as the mean ± SD, with two replicates for Figure S2J and three replicates for Figure S2K. (L and M) Maize seedlings were pretreated for two days under different light conditions (80 lux for Red and White light), and then the detached leaves were used for dynamic monitoring of chitin-elicited ROS. The data are represented as the mean ± SD (*n* = 8). Experiment was performed three times, with results of one representative experiment shown. The means of each time point were analyzed using a two-tailed paired Student’s *t-*test to determine the statistical significance of the observed differences in comparison to the white condition. Scale bars in Figure S2A,B,I represent 1 cm. Different letters indicate significant difference between treatments in Figure S2E‒H (lowercase letter: *p* < 0.05; uppercase letter: *p* < 0.01).


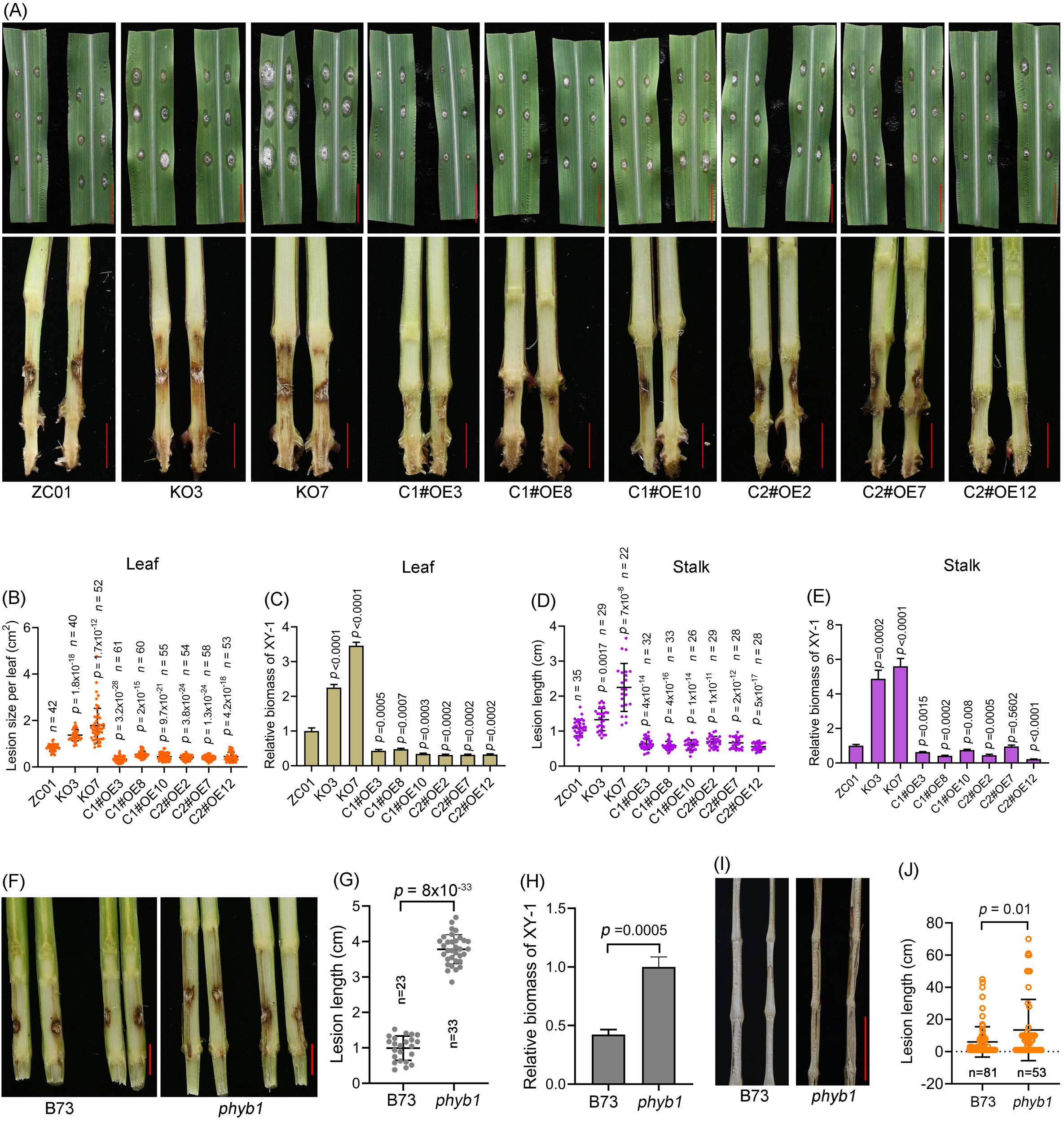


**Figure S3 *ZmPHYC*s and *ZmPHYB1* genes are important for resistance to *F. verticillioides* invasion in maize.** (A‒H) Thirty-day-old maize seedlings grown under normal conditions (4000 lux) were exposed to *F. verticillioides*, and the leaf and stalk resistance were investigated, respectively. Then, leaf lesion size, stalk lesion length, and the biomass of *F. verticillioides* in the corresponding infected tissues were investigated. (A‒E) KO3 and KO7 are two lines of *phyc1 phyc2* double mutants in the ZC01 genetic background. C1#OE3, C1#OE8, and C1#OE10 are three lines of *PHYC1*-overexpressing transgenic plants also in ZC01 background. Similarly, C2#OE2, C2#OE7, and C2#OE12 are three lines of *PHYC2*-overexpressing transgenic plants in the same background. ZC01 represents the segregated wild type from the transgenic plants. Scale bar = 1 cm. (I and J) Field observation of stalk rot in B73 and *phyb1* mutants caused by *F. verticillioides*. Since the genetic background of the *phyb1* mutant is B73, B73 was used as the wild-type control in Figure S3F‒J. Scale bar = 10 cm. The data are represented as the mean ± SD. Three biological replicates were performed for S3A, and two biological replicates were performed for S3F and S3I, respectively, with results of one representative experiment shown. Statistical analysis was performed using unpaired Student’s *t-*test (two tailed).


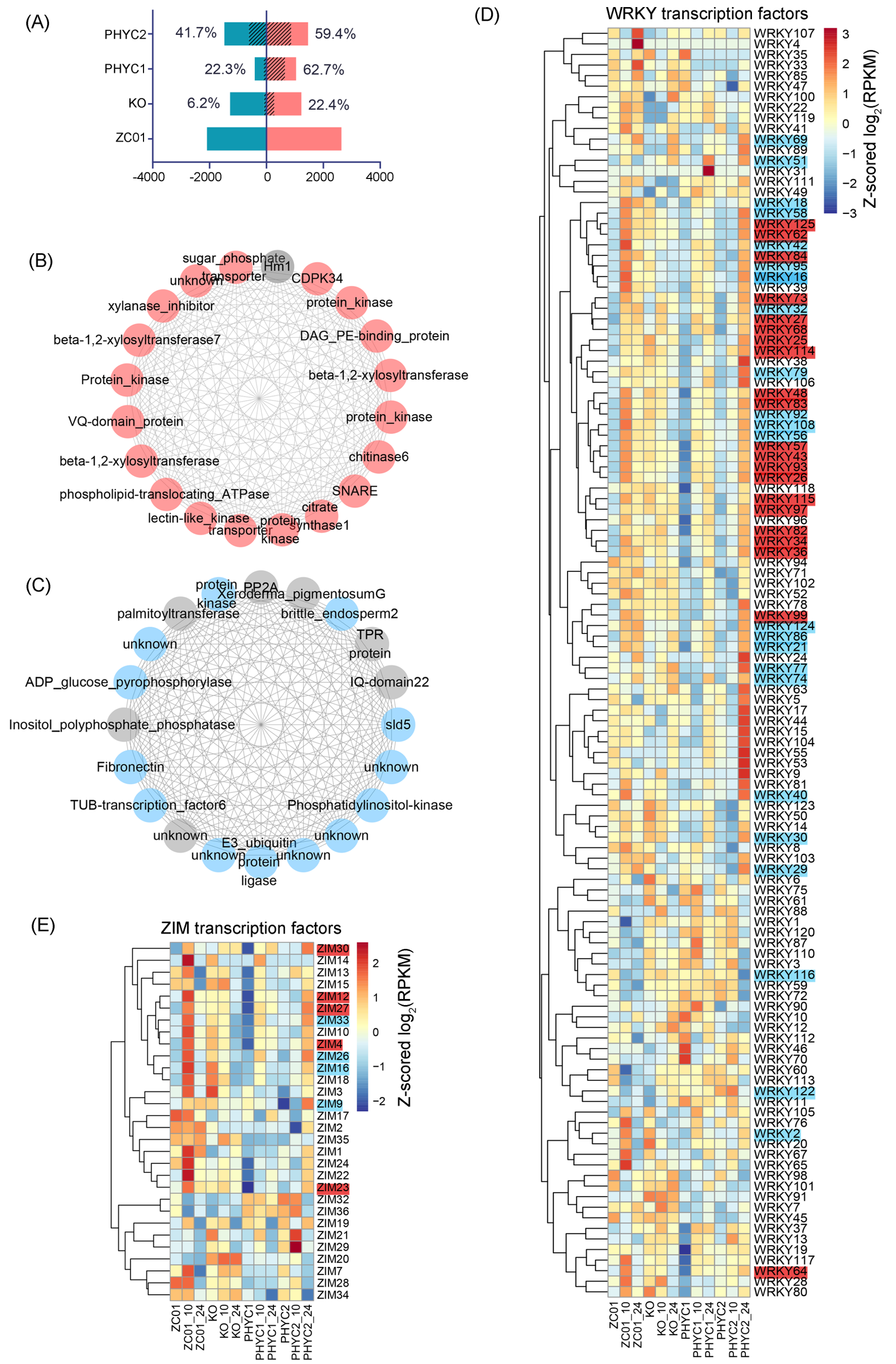


**Figure S4 WRKY and ZIM transcription factors are enriched in the PHYC-dependent defense gene module.** (A) The number of down-regulated (blue histograms) and up-regulated genes (red histograms) under *F. verticillioides* infection in the overexpressing plants (PHYC1#OE3, PHYC2#OE12), knockout mutants (the *phyc1 phyc2* double mutants KO#7), and wild-type (ZC01) are presented. The overlapping parts between the overexpressing plants or knockout mutants and the wild-type ZC01 are denoted with slashes, along with their corresponding proportions. (B and C) The top 20 genes with the highest correlation with eigengenes in the blue and turquoise modules were visualized in two networks using Cytoscape. Genes marked in pink or blue belong to UPDGs and DPDGs, respectively. Genes marked in gray do not belong to either group. (D and E) Heatmaps depicting the transcription levels of all detected *ZIM*s and *WRKY*s across different samples. Genes highlighted in red are part of the intersection between the blue module and UPDGs, while those marked in blue are exclusive to the blue module.


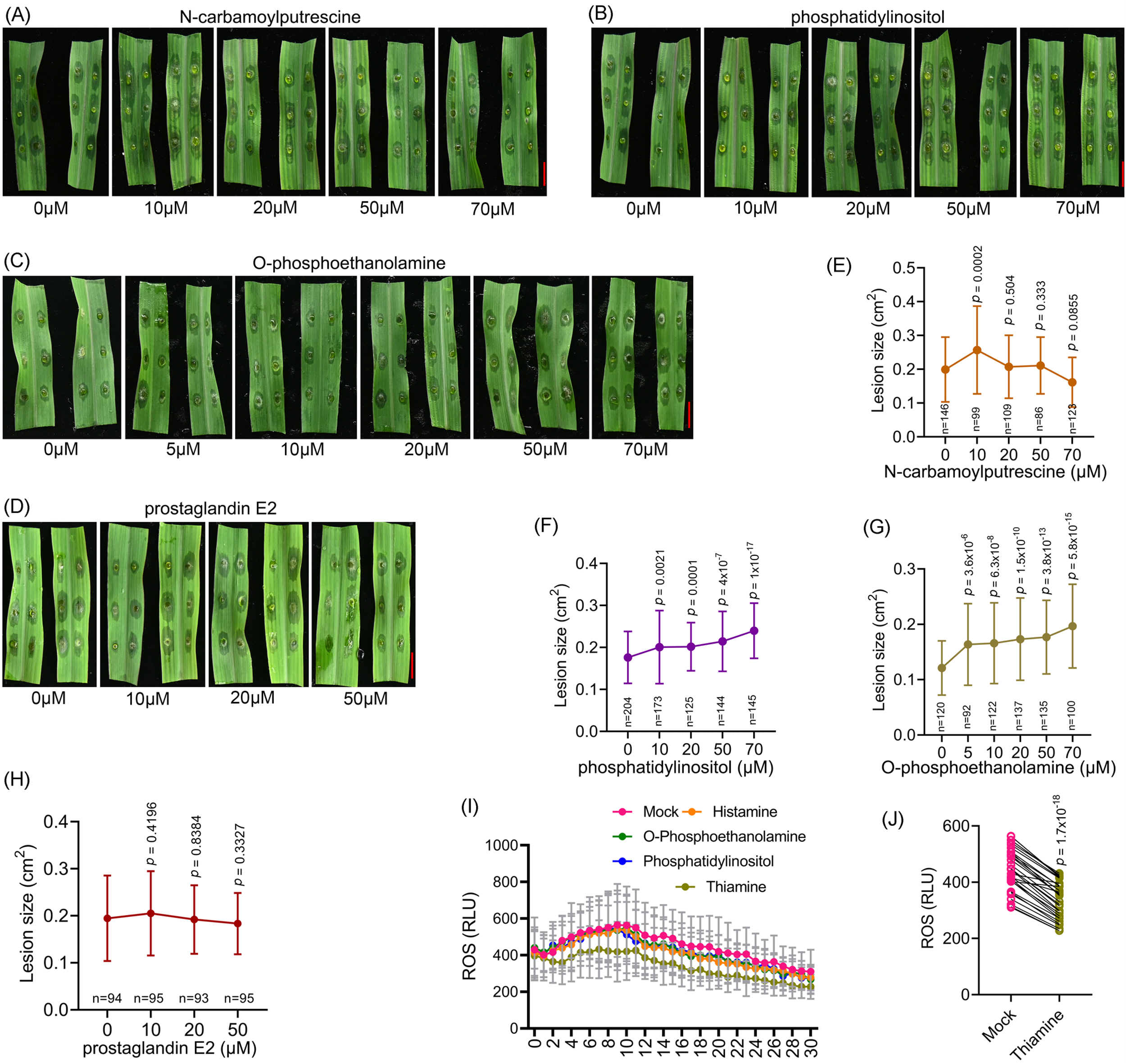


**Figure S5 O-phosphoethanolamine and phosphatidylinositol decrease maize resistance to *F. verticillioides.*** Detached leaves were pretreated with (A and E) N-carbamoylputrescine, (B and F) phosphatidylinositol, (C and G) O-phosphoethanolamine, and (D and H) prostaglandin E2 at different concentrations for 6 h, followed by exposure to *F. verticillioides* for two days to assess lesion size. The data are represented as the mean ± SD, from three replicates. (I and J) Maize seedlings were pretreated with histamine (50 μM), thiamine (20 mM), phosphatidylinositol (50 μM), and O-phosphoethanolamine (50 μM) for 6 h, after which detached leaves were used for dynamic monitoring of chitin-elicited reactive oxygen species. The data are represented as the mean ± SD (*n* = 40), from five replicates. The means of each time point were used for a paired Student’s *t-*test (two tailed) to ascertain the statistical significance of the observed differences between different treatments. The thiamine-treated group exhibited a significant difference compared to the control group.


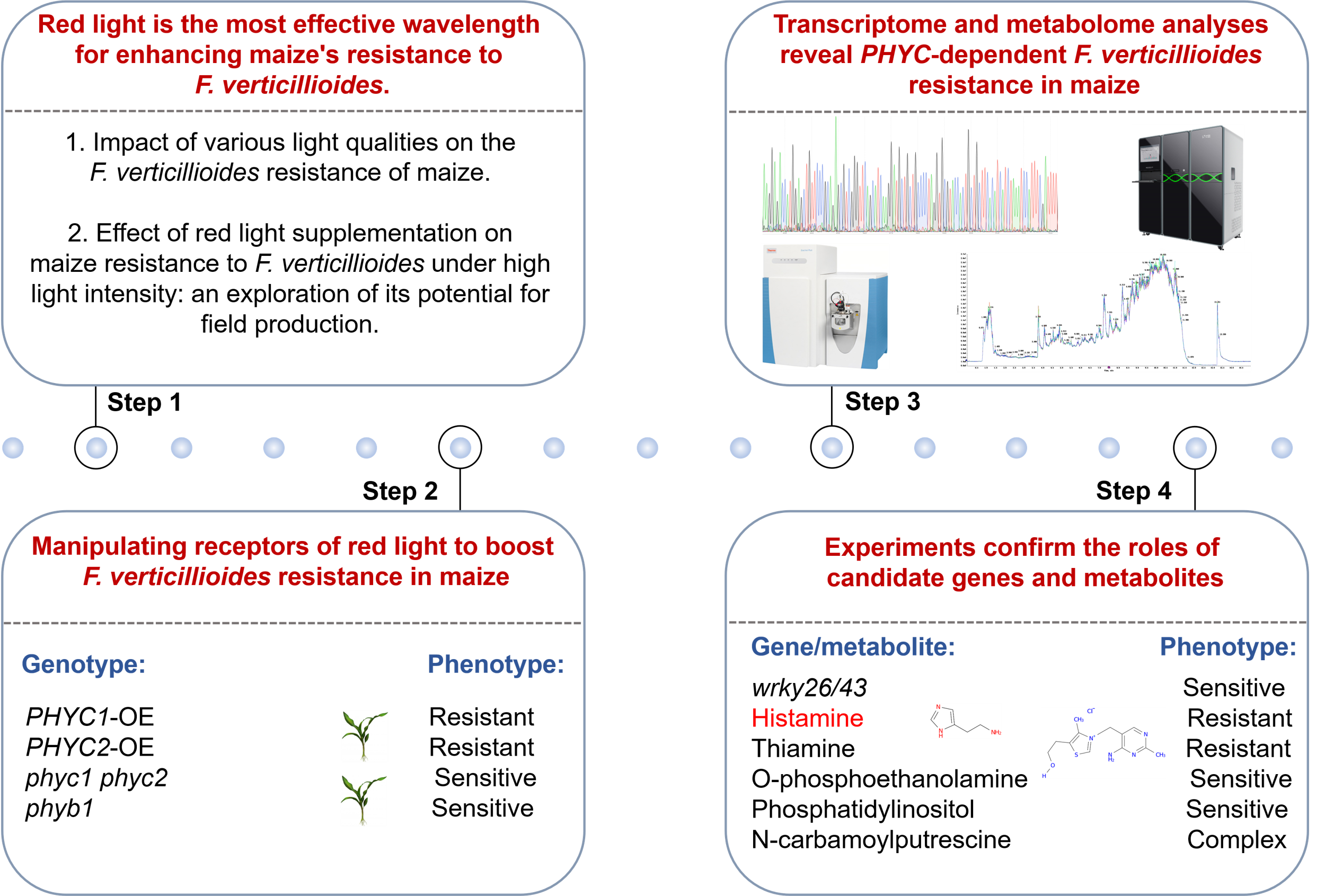


**Figure S6 Framework of this study.**


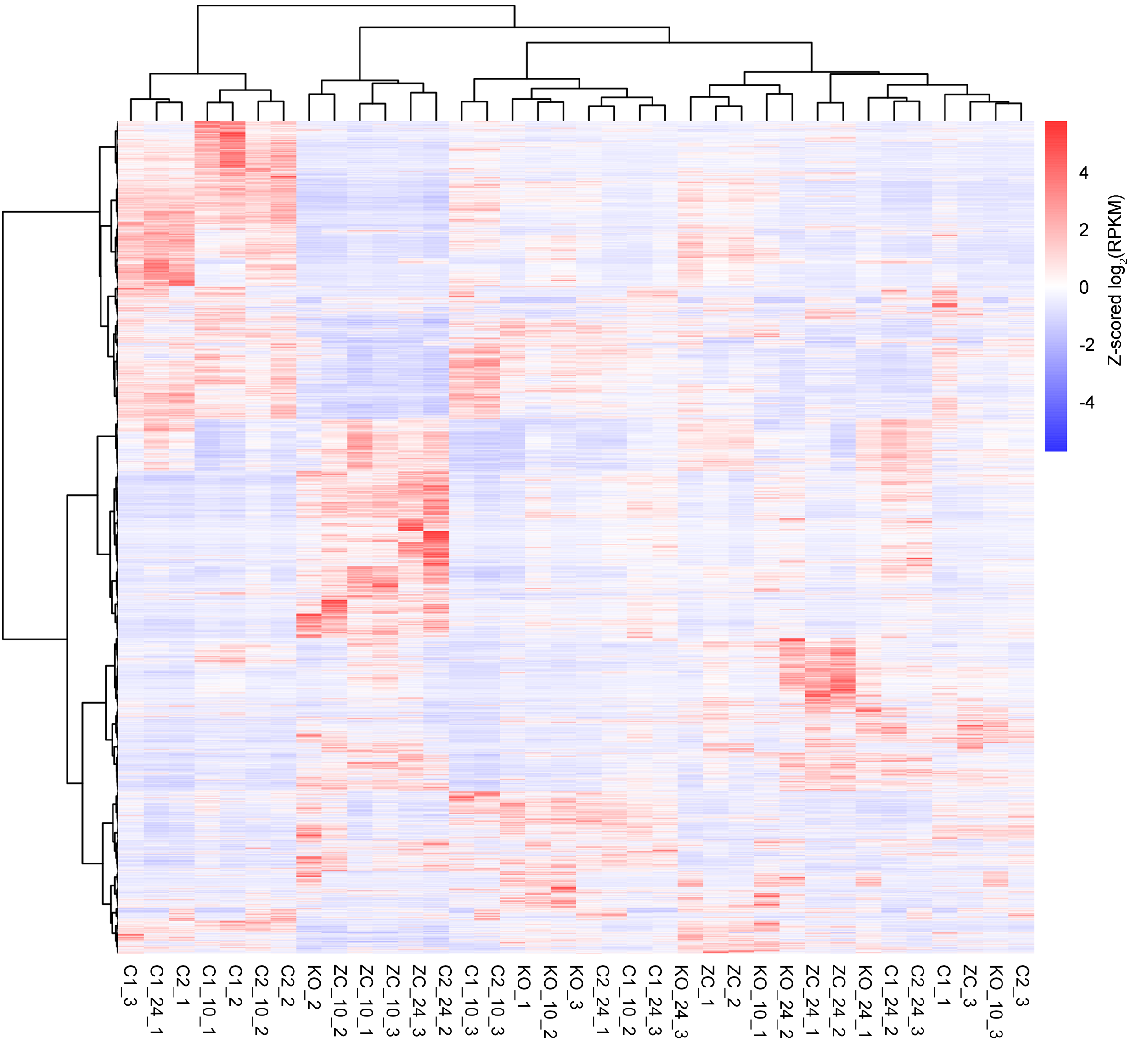


**Figure S7 Clustering heatmaps of the transcriptomic data of *PHYC1* overexpressing plants, *PHYC2* overexpressing plants, *phyc1 phyc2* double mutants, and the wild-type ZC01.**


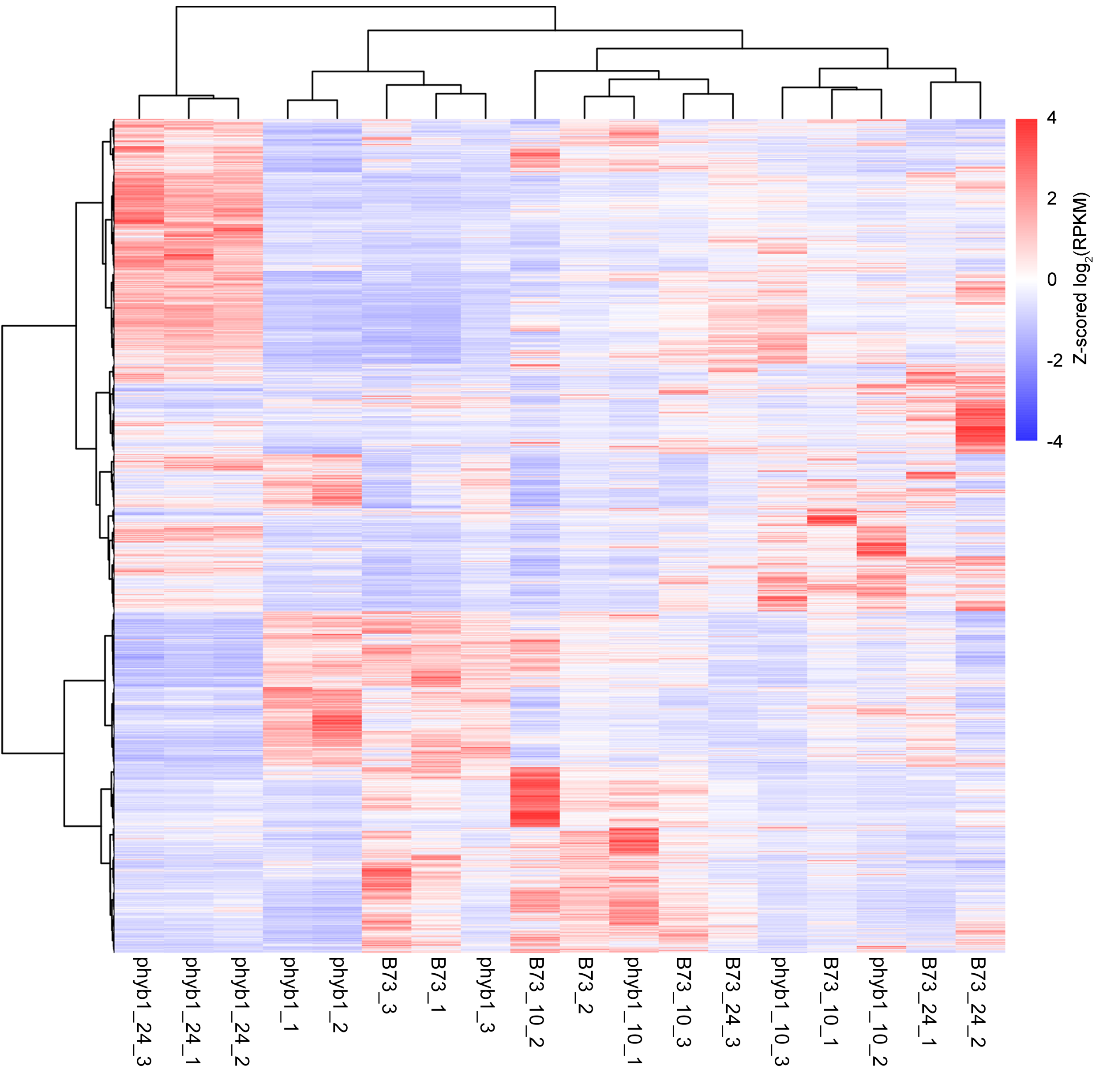


**Figure S8 Clustering heatmaps of the transcriptomic data of *phyb1* and the wild-type B73.**


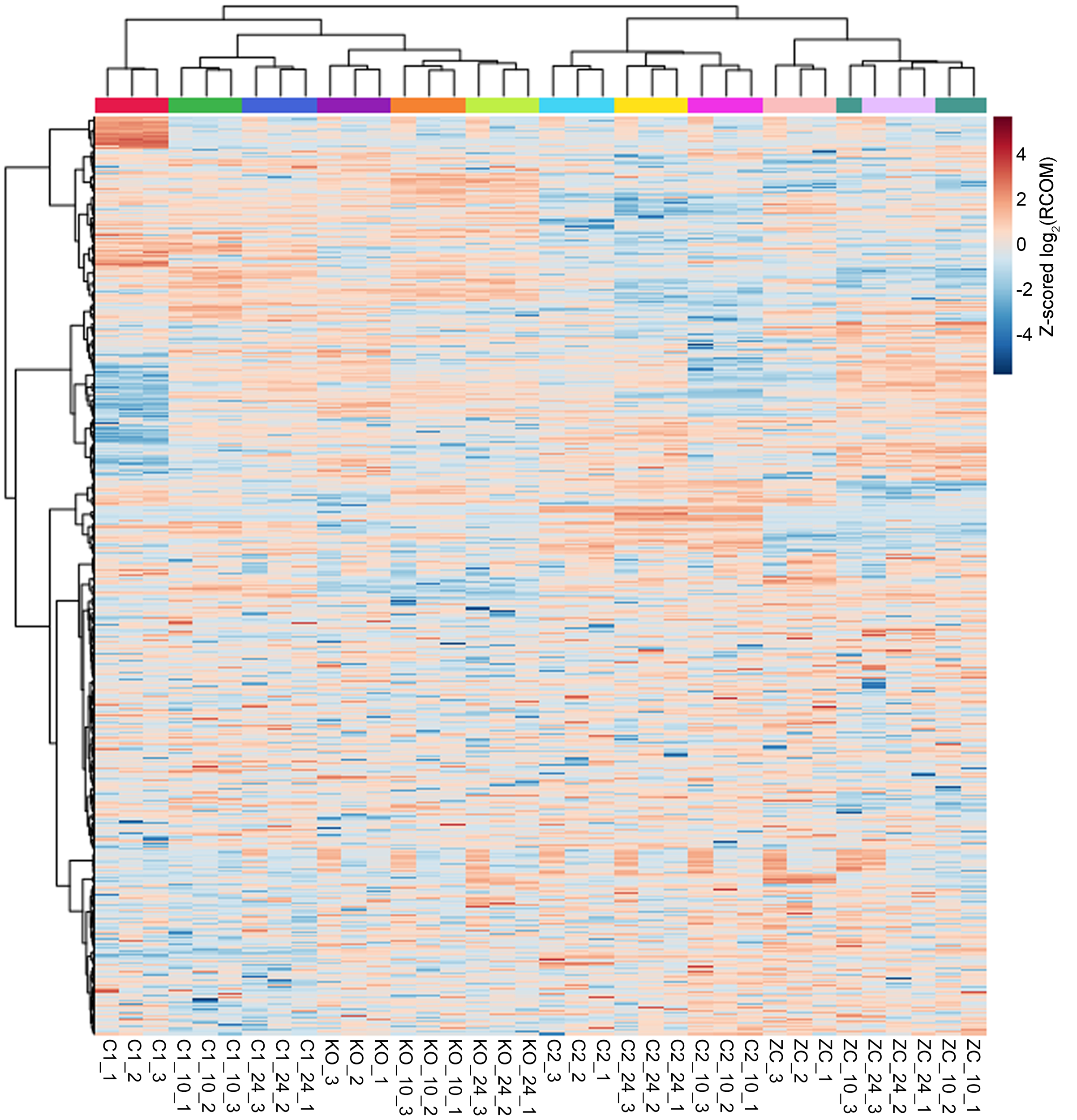


**Figure S9 Clustering heatmaps of the metabolomic data of *PHYC1* overexpressing plants, *PHYC2* overexpressing plants, *phyc1 phyc2* double mutants, and the wild-type ZC01. RCOM indicates the relative content of metabolites.**


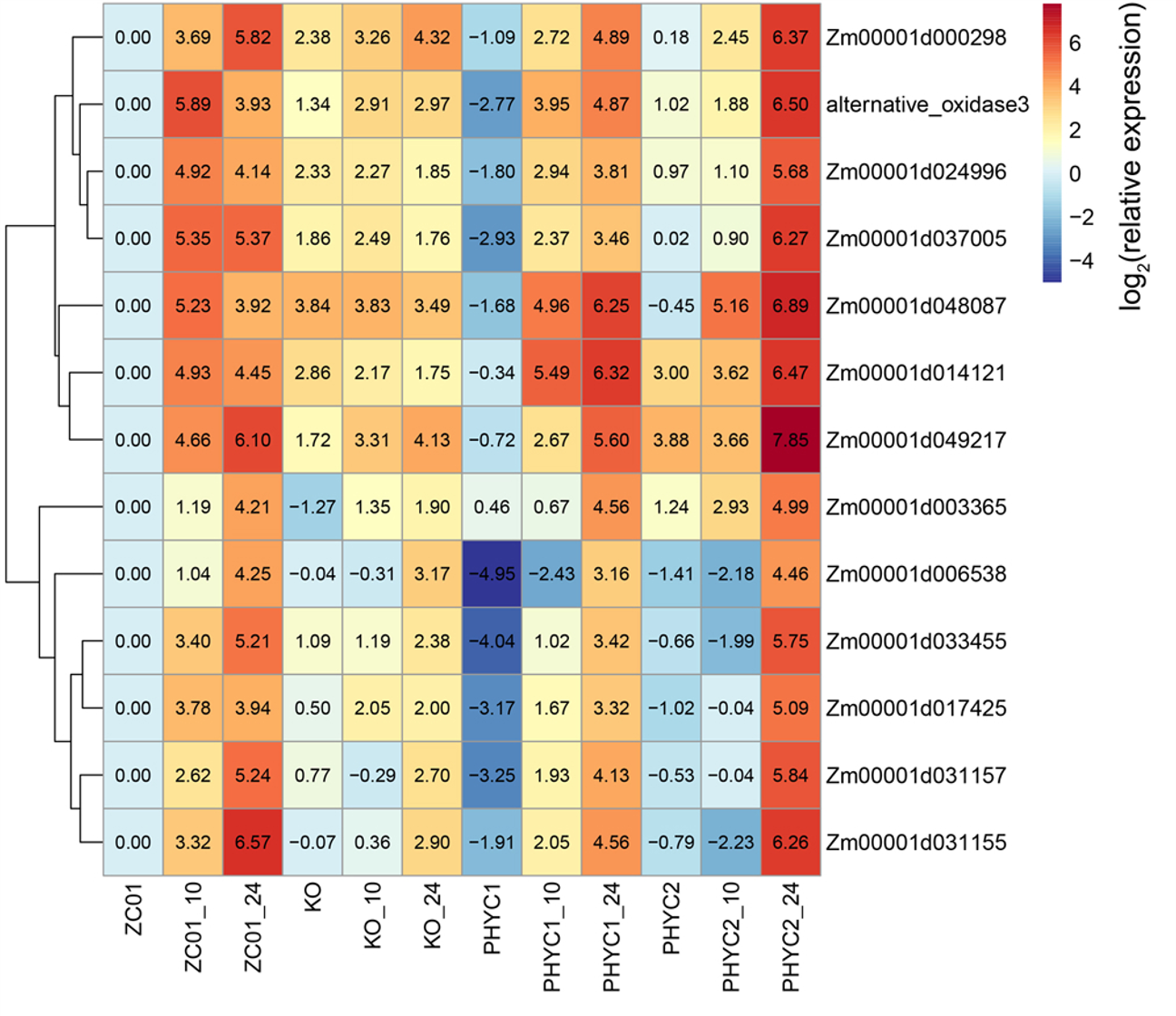


**Figure S10 Ten genes with different expression levels were selected to validate the RNA-seq results.** The relative expression levels were calibrated to ZC01 and presented as a heatmap using log2-transformed values. 18S rRNA and *ZmEF1a* were used as reference genes to normalize the expression levels of candidate genes.
